# Editorial: Pain 360: Emerging topics in the pathophysiology, diagnosis, and treatment of chronic pain

**DOI:** 10.3389/fpain.2022.1123272

**Published:** 2023-01-10

**Authors:** Craig T. Hartrick, Jose De Andres

**Affiliations:** ^1^ Oakland University, Rochester, MI, United States; ^2^ Department of Surgical Specialties, School of Medicine, General University Hospital, University of Valencia, Valencia, Spain

**Keywords:** pain phenotypes, biomarkers, spinal anatomy, spinal intervention, interdisciplinary pain investigation

**Editorial on the Research Topic** Pain 360: Emerging topics in the pathophysiology, diagnosis, and treatment of chronic pain

Pain 360 refers to a holistic, comprehensive 360-degree view of pain. The aim of the Pain 360 collaboration is to further foster interdisciplinary discussion and cross-fertilization to promote innovation. The goal of Pain 360 is specifically not to create yet another pain society. Rather it is to bring together discussants from all relevant pain and pain-related societies, as well as other important stakeholders, including patients and caregivers, to engage in constructive conversation.

In a world in which science advances and knowledge increases exponentially, there is a necessary and increasing trend toward the creation of societies that represent a specialized portion of that knowledge, thus facilitating deeper dives into specific areas. Accordingly, knowledge relating to the investigation and treatment of chronic pain has advanced in a spectacular way in recent years. However, it has become clear that alongside these efforts to “miniaturize” knowledge, a holistic vision is essential to allow the integration of all key elements, with the patient taking center stage in our performance. The possibility of a 360° vision, without ties or constraints, from basic science to clinical practice, always considering the translation to the human condition, is the motivation of this proposal for interactive professional discussion.

Frontiers in Pain Research, with its multifaceted Editorial Board structure covering nearly every aspect of the pain field, is a particularly appropriate vehicle for this endeavor. We are very grateful for the opportunity to present this initial offering on this platform.

While the original intention of this Research Topic was to highlight some presentations from a Pain 360 congress in Vienna, the in-person event was cancelled due to the COVID-19 shutdown. Several of the articles presented here are in part based on concepts that were to be presented at that meeting. Nevertheless, the discussion has continued virtually with, as of this writing, two webinars focusing on Pain Measurement. We hosted these sessions along with our other collaborator, Michael Gofeld, MD, PhD. The second webinar included Michelle M. Langer, MD, discussing PROMIS (Patient-Reported Outcomes Measurement Information System) and Michael Fishman, MD, discussing the use of PROMIS in both patient-centered research and clinical care. Based upon feedback from these sessions, which included discussants with widely varied backgrounds and perspectives from over 80 countries, the next sessions are being planned.

This Research Topic in Frontiers in Pain Research introduces some emerging concepts that aim to provide a mechanistic bridge between basic and clinical pain science. The first is Hartrick's brief opinion piece discussing the unmet need for new analgesics and the difficulty in bringing novel molecules to market. Attempts to overcome some of the inherent difficulties with typical animal model testing through understanding and exploiting pathophysiologic changes and then using biomarkers that identify candidates for therapy based upon those changes is discussed. Other innovative lines of research that likewise may improve the translation to humans, and thus improve the success rate for analgesic development, are actively being pursued and represent fertile ground for ongoing discussion. These include the use of *ex vivo* human tissue to gain further insight into both efficacy (1) and safety (2) prior to clinical trials.

De Andres et al. next present a comprehensive review of intrathecal drug delivery. The safe and effective management of these systems relies upon a sound understanding of the anatomy and physiology of the spinal cord, its surrounding structures, and the dynamics of cerebrospinal fluid production and circulation in humans. Up-to-date imaging techniques demonstrate features relevant to treatment success, optimal placement of the devices and dosing, as well as potential complications in this dynamic environment. Yet even in the arena of gross anatomy, there remain unanswered questions. Further research into the often-overlooked peridural membrane and its true function represent related areas for further study (3, 4). The original research by Bosscher mathematically models the fluid dynamics following pressurized injection into the epidural space. These novel findings coupled with clinical experience are used to derive important safety recommendations in addition and complementary to those described by the preceding presentation.

In the final original research article, Robayo et al. present results from a cluster analysis of a battery of psychometric tests and quantitative sensory testing findings in patients with traumatic brain injury (TBI). The authors were able to identify specific pain phenotypes within their study population that might guide individualized or personalized treatment tailored to TBI patients. These clinical findings might also serve to assist in back-translation, selection of appropriate biomarkers, and the refinement of animal models to more closely mimic the human condition, bringing us full circle (360 degrees), back to the concepts alluded to previously.
